# Serum Testosterone Levels in 3-Month-Old Boys Predict Their Semen Quality as Young Adults

**DOI:** 10.1210/clinem/dgac173

**Published:** 2022-03-22

**Authors:** Louise Scheutz Henriksen, Jørgen Holm Petersen, Niels E Skakkebæk, Niels Jørgensen, Helena E Virtanen, Lærke Priskorn, Anders Juul, Jorma Toppari, Katharina M Main

**Affiliations:** Department of Growth and Reproduction, Copenhagen University Hospital – Rigshospitalet, DK-2100 Copenhagen, Denmark; International Centre for Research & Training in Endocrine Disruption of Male Reproduction & Child Health (EDMaRC), Copenhagen University Hospital – Rigshospitalet, DK-2100 Copenhagen, Denmark; Department of Growth and Reproduction, Copenhagen University Hospital – Rigshospitalet, DK-2100 Copenhagen, Denmark; International Centre for Research & Training in Endocrine Disruption of Male Reproduction & Child Health (EDMaRC), Copenhagen University Hospital – Rigshospitalet, DK-2100 Copenhagen, Denmark; Section of Biostatistics, Department of Public Health, University of Copenhagen, DK-1353 Copenhagen, Denmark; Department of Growth and Reproduction, Copenhagen University Hospital – Rigshospitalet, DK-2100 Copenhagen, Denmark; International Centre for Research & Training in Endocrine Disruption of Male Reproduction & Child Health (EDMaRC), Copenhagen University Hospital – Rigshospitalet, DK-2100 Copenhagen, Denmark; Department of Clinical Medicine, Faculty of Health Sciences, University of Copenhagen, DK-2200 Copenhagen, Denmark; Department of Growth and Reproduction, Copenhagen University Hospital – Rigshospitalet, DK-2100 Copenhagen, Denmark; International Centre for Research & Training in Endocrine Disruption of Male Reproduction & Child Health (EDMaRC), Copenhagen University Hospital – Rigshospitalet, DK-2100 Copenhagen, Denmark; Research Centre for Integrative Physiology and Pharmacology, Institute of Biomedicine, University of Turku, 20520 Turku, Finland; Centre for Population Health Research, University of Turku and Turku University Hospital, 20520 Turku, Finland; Department of Growth and Reproduction, Copenhagen University Hospital – Rigshospitalet, DK-2100 Copenhagen, Denmark; International Centre for Research & Training in Endocrine Disruption of Male Reproduction & Child Health (EDMaRC), Copenhagen University Hospital – Rigshospitalet, DK-2100 Copenhagen, Denmark; Department of Growth and Reproduction, Copenhagen University Hospital – Rigshospitalet, DK-2100 Copenhagen, Denmark; International Centre for Research & Training in Endocrine Disruption of Male Reproduction & Child Health (EDMaRC), Copenhagen University Hospital – Rigshospitalet, DK-2100 Copenhagen, Denmark; Department of Clinical Medicine, Faculty of Health Sciences, University of Copenhagen, DK-2200 Copenhagen, Denmark; Research Centre for Integrative Physiology and Pharmacology, Institute of Biomedicine, University of Turku, 20520 Turku, Finland; Centre for Population Health Research, University of Turku and Turku University Hospital, 20520 Turku, Finland; Department of Pediatrics, Turku University Hospital, 20520 Turku, Finland; Department of Growth and Reproduction, Copenhagen University Hospital – Rigshospitalet, DK-2100 Copenhagen, Denmark; International Centre for Research & Training in Endocrine Disruption of Male Reproduction & Child Health (EDMaRC), Copenhagen University Hospital – Rigshospitalet, DK-2100 Copenhagen, Denmark; Department of Clinical Medicine, Faculty of Health Sciences, University of Copenhagen, DK-2200 Copenhagen, Denmark

**Keywords:** male reproductive health, semen quality, minipuberty, hypothalamic-pituitary-gonadal (HPG) axis

## Abstract

**Context:**

It remains unknown how the postnatal activation of the hypothalamic-pituitary-gonadal axis in infancy, also known as “minipuberty”, relates to adult testis function.

**Objective:**

To investigate how markers of reproductive function in 3-month-old boys correlate with adult reproductive health parameters.

**Methods:**

This population-based birth cohort study (the Copenhagen Mother-Child cohort), conducted at Copenhagen University Hospital, Denmark, included 259 boys examined once around 3 months of age and again at 18 to 20 years. Reproductive hormones, penile length, testis volume, and semen quality were analyzed. Minipubertal markers of testis function (by tertiles, T1–T3) were explored as predictors of adult semen quality using linear regression models. Associations between reproductive outcomes in infancy and young adulthood were estimated by intraclass correlation coefficients (ICCs), describing how well measurements in infancy correlate with those in adulthood.

**Results:**

Serum testosterone concentration in infancy was positively associated with adult total sperm count. Median (IQR) total sperm count was 84 (54-138) million spermatozoa for boys in T1, 141 (81-286) million spermatozoa in T2, and 193 (56-287) million spermatozoa in T3. We found the highest ICC for FSH (0.41; 95% CI, 0.26–0.57), while ICCs for inhibin B, SHBG, penile length, and testis volume ranged between 0.24 and 0.27. ICCs for LH and for total and free testosterone were lower and statistically nonsignificant.

**Conclusion:**

Serum testosterone in infancy was a predictor of adult total sperm count. Other reproductive hormones and genital measures showed good correlation between infancy and adulthood, suggesting that an individual’s reproductive setpoint starts shortly after birth in boys and persists until adulthood.

The term *minipuberty*, first described in the 1970s (1), denotes a period shortly after birth during which a transient activation of the hypothalamic-pituitary-gonadal (HPG) hormone axis is evidenced. The role of this postnatal activity of the gonads is not known. Neither has it been established how testosterone concentrations and other markers of testicular function in early childhood are connected to reproductive functions in adulthood.

Experimental research has suggested that programming of adult reproductive function occurs early during fetal life. Disturbed development may cause permanent damages resulting in disruption of genital development (2). Preclinical data are in line with human data showing that maldevelopment of the testis is associated with congenital malformations of the male genitalia, for example, undescended testis and hypospadias, and also a risk of adult reproductive problems, such as testicular cancer and infertility (3, 4).

In this exploratory study, we hypothesized that the HPG axis activation occurring during infancy is a “window of opportunity” in healthy infant boys that may predict adult testicular function. We therefore followed a large group of boys from birth to young adulthood and examined whether pituitary and gonadal hormone levels at 3 months of age were associated with reproductive functions, including semen quality, at 18 to 20 years of age.

## Methods

### Study Design and Participants

This study is a prospective population-based birth cohort study as previously described (5). The cohort was established 1997–2001 with consecutive recruitment of healthy pregnant women in their first trimester. A total of 1270 of their live-born sons were included and invited for repetitive examinations after birth and again in young adulthood at 18 to 20 years. For the adult examination, young men who had participated in at least one perinatal examination were invited (1078 young men were invited, 265 (25%) participated). In the current study, we included 260 sons who participated in the 3-month examination (1997–2001) and adult examination (2018–2019). One young man with azoospermia, who did not have a postnatal serum sample, was excluded. Data from early infancy in this cohort have previously been published (5-9). The study was performed following the Declaration of Helsinki II. Both examinations were approved by the Regional Committee on Health Research Ethics ([KF] 01-030/97 and H-17011468) and the Danish Data Protection Agency (1997-1200-074 and VD-2018-118/i-Suite 6358). All participants gave written informed consent.

### Study Setup

#### Key outcomes

Reproductive outcomes in both infancy and adulthood comprised penile length, mean testicular volume, and serum concentrations of reproductive hormones. In adulthood, semen quality variables were also included (total sperm count [million], progressively motile spermatozoa [%], and morphologically normal spermatozoa [%]).

#### Pregnancy and birth information

Information on gestational age (GA) at birth was based on routine ultrasound investigation in pregnancy weeks 18 to 20 if available, or the last menstrual period (5). The boys were defined as preterm (GA < 37 weeks), full-term (37 weeks ≤ GA ≤ 42 weeks), or post-term (GA > 42 weeks). Birth weight was obtained from birth records (7), and weight for gestational age (WGA) was expressed as the deviation from the expected mean in percentage. Small for gestational age (SGA) was defined as WGA below −22% (−2 SD) and large for gestational age (LGA) as WGA above 22% (+2 SD). Information on maternal parity, smoking during pregnancy, and gestational diabetes was obtained from hospital records.

#### Examination in infancy

Infant boys were examined around 3 months of age, and in case of preterm birth the examination was timed according to expected day of delivery (5). Using an antecubital vein, a nonfasting blood sample was drawn between 09:00 and 16:00 hours. Testicular length and width were measured 3 times using ultrasonography, and the formula length × width^2 ^× (π/6) was used to calculate testicular volume (mm^3^) (9). An average of 3 calculations was used. The mean volume of left and right testis was calculated and converted to milliliters (dividing by 1000). Using a slide gauge (Baty International, Burgess Hill, West Sussex, UK), the distance between the lower edge of the pubic bone and the tip of the glans penis (excluding foreskin) was measured after slightly straightening the flaccid penis (8).

#### Adult examination

An online questionnaire, including questions on smoking habits, was completed at home before the examination. Blood samples were obtained from an antecubital vein between 07:30 and 12:00 hours in a fasting state. The young men delivered a semen sample by masturbation in a designated room at the department. Sexual abstinence for more than 48 hours before sampling was recommended, and the exact period of abstinence was recorded. The length of the penis (flaccid, vertically adjusted) was measured to the nearest millimeter using a ruler with the participant in a supine position. Testicular length, width, and height were measured 3 times using ultrasonography, and testis volume (mm^3^) was calculated using the formula height × length × width × (π/6) (10). An average of 3 calculations was used. The mean volume of left and right testis was calculated and converted to milliliters (dividing by 1000). Participants received DKK 500 (≈€67) as compensation for their time. All participants also underwent a whole-body dual-energy x-ray absorptiometry (DXA) scan for assessment of total body fat percentage (Lunar Prodigy, GE Healthcare, Madison, WI, using enCORE software, version 14.10.022).

### Hormone Assays

Blood samples were centrifuged and stored at −20 °C until analysis. Analyses of hormones were performed in our accredited laboratory (Rigshospitalet, Copenhagen). The analyses were accredited by the Danish Accreditation Fund for medical examination according to a Danish approved European and International standard (the standard DS/EN ISO 15189). Methods for hormone analyses in infant boys have previously been described (7). In both infant boys and young men, serum levels of follicle-stimulating hormone (FSH) and luteinizing hormone (LH) were measured using 2-sided time-resolved immunofluorometric assays (AutoDELFIA, Perkin Elmer, FSH: Cat# B017-201, RRID: AB_2783738; LH: Cat# B031-101, RRID: AB_2783737). Serum inhibin B concentrations were measured by an enzyme-linked immunosorbent assay (ELISA) of 2 different types (infant boys: Oxford Bio-Innovation, Cat# MCA1312KZZ, RRID: AB_2800544; young men: inhibin B gen II, Beckman Coulter, Cat# A81303, RRID: AB_2827405). In infant boys, 2 different radioimmunoassays (RIAs) were used to measure serum concentrations of estradiol (Pantex, Cat# 174M, RRID: AB_2905658) and testosterone (Coat-a-Count, Siemens, Cat# TKTT5, RRID: AB_2905660). In the young men, these 2 hormones were measured by a chemiluminescent enzyme immunoassay (Access2, Beckman Coulter, testosterone: Cat# 33560, RRID: AB_2905661; estradiol: Cat# B84493, RRID: AB_2905662). Serum sex hormone binding globulin (SHBG) in young men was also measured by Access2 (Beckman Coulter, Cat# A48617, RRID: AB_2893035), while it was measured using the AutoDELFIA immunofluorometric assay in infant boys (Perkin Elmer, Cat# B070-101, RRID: AB_2905659). Internal validation programs were conducted with every assay change to standardize the analyses (data not shown). All limits of detections (LODs) and inter-assay coefficients of variation (CVs) are reported in Supplemental Table 1 (11).

At both ages, free testosterone was calculated using the equation by Vermeulen (12) (serum albumin was available in the young men while a fixed level of 40 g/L albumin was used for calculations in infancy). Ratios between free testosterone/LH, total testosterone/LH, and inhibin B/FSH were calculated by simple division. Out of 151 young men who had a serum estradiol measurement in early infancy and adulthood, only 71 (53.6%) had a level above the LOD in infancy; therefore, we excluded estradiol from further analyses.

### Semen Analyses

Semen samples were placed at 37 °C in an incubator immediately after being received in the laboratory. Analysis was initiated within 1 hour after sampling. Analyses were performed in accordance with the World Health Organization’s 2010 guidelines (13). Semen volume was determined by weighing, while sperm concentration was assessed using a NucleoCounter NC-3000 (ChemoMetec A/S, Alleroed, Denmark). Manual counts were performed in young men with very low concentrations (<3 million/mL) in accordance with our published procedures (14). Total sperm count was calculated by multiplication of semen volume with sperm concentration. For assessment of sperm motility, 2 drops of well-mixed semen were placed on a glass slide and examined with phase-contrast microscopy. Spermatozoa were classified as progressively motile, nonprogressively motile, or immotile. Fixed and Papanicolaou stained morphology slides were evaluated according to Krüger’s strict criteria (15) to determine the number of morphologically normal spermatozoa. Counts were done in duplicates, and the averages were used in statistical analyses. To standardize analyses, internal validation programs were conducted (data not shown).

### Statistics

Descriptive statistics on reproductive characteristics, lifestyle, and pregnancy/birth outcomes were calculated for both nonparticipants and participants in the adult follow-up. Group differences were tested using Kruskal-Wallis rank-sum test (continuous variables), chi-squared test of independence (categorical variables, expected cell counts ≥ 5), and Fisher’s exact test (categorical variables, expected cell counts < 5).

In exploratory analyses, adult semen parameters were compared between infant reproductive outcome tertiles (lower tertile = T1, middle tertile = T2, upper tertile = T3) by regression models using tertiles as categorical variables. This was done to explore potential nonlinear associations. To evaluate a potential dose-response effect, linear regression analyses were also performed using infant tertiles as continuous variables (p_trend_). Total sperm count by infant testosterone tertiles was illustrated using boxplots, and differences were tested with the Wilcoxon rank-sum test.

For all reproductive markers obtained both at the infant and adult examination, we calculated z-scores for each individual by internal standardization using the normal distribution quantile function evaluated in the empirical distribution function. Intraclass correlation coefficients (ICCs) were calculated for z-scores using a linear mixed model in which inter- and intra-individual variation were estimated. ICC is the fraction of the total variation of an outcome explained by the variation of the individual and measures how well the first measured outcome correlates with the later measured outcome. Confidence intervals (CIs) were estimated using the equivalence of the ICC and a regression parameter in a covariance analysis regressing adult z-score on the infant z-score. Negative values in CIs were replaced by zero, that is, CIs including zero were nonsignificant.

Both linear regression and ICC calculations were conducted unadjusted and after adjusting for maternal smoking (yes, no) and diabetes (yes, no), GA at birth, WGA, postnatal age at the minipuberty exam, smoking habits (nonsmoker, occasionally, daily) and total body fat percentage of the young men. In analyses including reproductive hormones, time of day for serum sampling was included. In analyses of semen quality, duration of abstinence was included and for sperm motility furthermore time passed between ejaculation and motility analysis. Linear regression model assumptions were checked using residual plots. To comply with the assumptions, total sperm count was square-root–transformed.

We repeated all analyses in a subgroup of the study population including only full-term boys born appropriate for gestational age (AGA) or LGA with an infant exam at 2.5 to 3.5 months of age and no cryptorchidism at birth or the 3-month examination (n = 194). Two observations for key outcomes were identified as outliers, and analyses were repeated after exclusion of these. As this did not substantially change the results, they were included in final analyses.

We adjusted all *P* values from fully adjusted models according to the Benjamini-Hochberg method (16, 17). All reproductive hormone values < LOD were replaced with LOD/√2. We considered *P* values < 0.05 as statistically significant. Statistical analyses were conducted using R software (R Core Team (2019). R: A language and environment for statistical computing. R Foundation for Statistical Computing, Vienna, Austria. URL https://www.R-project.org/). The “lme4” package was used to fit linear mixed-effects models, and figures were constructed using “ggplot2”. The “stats” package was used to obtain *P* values adjusted for multiplicity.

## Results

The median age at the minipubertal and adult exams was 3.1 months and 19.3 years, respectively (Table 1). Young men who participated in the adult follow-up did not differ from nonparticipants regarding maternal, birth, and minipubertal characteristics, except more mothers of nonparticipants smoked during pregnancy (30% vs 22%; *P* = 0.013) (Supplemental Table 2 (11)). Markers of testis function in infancy and adulthood are reported in Supplemental Table 3 (11).

**Table 1.** Characteristics of the study population

	n	Median (IQR); n (%)
**Adulthood**		
Age at adult examination, years	259	19.3 (18.9, 19.7)
Height, cm	259	183.9 (179.7, 188.1)
Weight, kg	259	73.2 (66.0, 81.2)
BMI, kg/m^2^	259	21.5 (19.8, 23.8)
Total fat percent by DXA	252	19.5 (16.6, 25.0)
Ejaculation abstinence, hours	253	61 (48, 84)
Testis volume by orchidometer, mL	236	25.0 (23.0, 26.5)
Cigarette smoking, n (%)	252	
None		134 (53%)
Occasionally		88 (35%)
Daily		30 (12%)
**Infancy**		
Age at infant examination, months	259	3.1 (2.8, 3.4)
Body length, cm	258	62.1 (60.7, 63.8)
Body weight, kg	259	6.5 (6.0, 7.0)
Cryptorchidism in infancy^a^, n (%)	258	1 (0.4%)
**Birth characteristics**		
Birth weight, kg	259	3.6 (3.3, 4.0)
GA at delivery, n (%)	259	
Preterm		11 (4.2%)
Full-term		220 (85%)
Post-term		28 (11%)
Weight for gestational age, n (%)	259	
SGA		9 (3.5%)
AGA		240 (93%)
LGA		10 (3.9%)
Cryptorchidism at birth^a^, n (%)	251	3 (1.2%)
**Maternal characteristics**		
Maternal age at delivery, years	259	30.8 (28.1, 33.2)
Maternal parity, n (%)	259	
1		177 (68%)
2		68 (26%)
≥3		14 (5.4%)
Maternal smoking during pregnancy, n (%)	255	56 (22%)
Maternal diabetes, n (%)	256	3 (1.2%)

Abbreviations: AGA, appropriate for gestational age; BMI, body mass index; DXA, dual x-ray absorptiometry; GA, gestational age; IQR, interquartile range; LGA, large for gestational age; SGA, small for gestational age.

^a^Testicular position was recorded after manipulation of the testis to the most distal position along the pathway of normal descent using firm but not forced traction (5).

### Associations Between Infant Markers of Testis Function and Semen Quality

**Figure 1.**
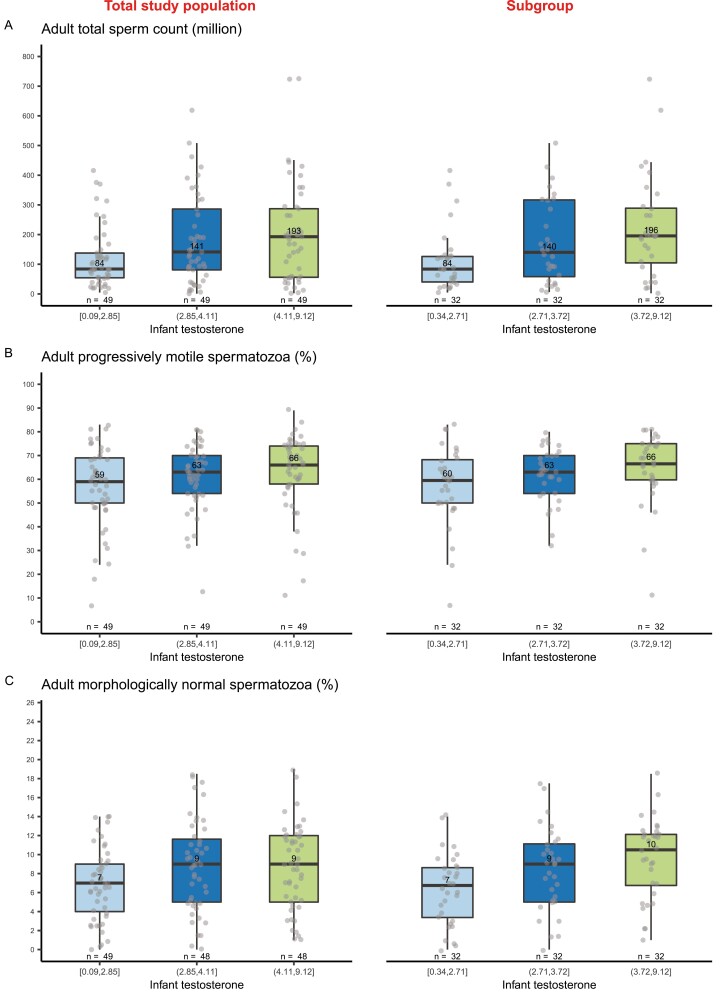
Semen quality outcomes by total testosterone tertiles in infancy. Analyses are presented for the total study population and a subgroup (full-term, not small for gestational age [SGA] or cryptorchid, and an examination at 2.5 to 3.5 months). The plots show (A) total sperm count (million), (B) progressively motile spermatozoa (%), and (C) morphologically normal spermatozoa (%) by total serum testosterone tertile in infancy in the total study population (left column, n = 147 [145 for morphology]) and a subgroup (right column, n = 96). Values shown are medians, lower quartile (Q1), upper quartile (Q3), upper whisker = maximum(x[x < Q3 + 1.5 × IQR]), and lower whisker = minimum(x[x > Q1 + 1.5 × IQR]), where IQR is the interquartile range. Filled circles are individual observations, and numbers inside boxplots are medians. Plot A: One outlier (total sperm count of 1065 million spermatozoa) in the middle tertile is not shown.

Overall, markers of the LH/Leydig cell axis in infancy were associated with semen parameters. The same conclusions were reached with both unadjusted and adjusted models. Higher testosterone in infant boys was associated with higher total sperm count in adulthood (Fig. 1, Table 2). While men with infant testosterone concentrations in the lowest tertile (T1) had a median total sperm count of 84 million (interquartile range [IQR], 54-138), total sperm count was 141 (81-286) and 193 (56-287), respectively, for boys with a testosterone in T2 and T3. This became more pronounced in the subgroup which included only full-term boys who were not SGA or cryptorchid, and who were examined 2.5 to 3.5 months after birth (Fig. 1, Table 2). There was also a positive association between testosterone in infancy and sperm motility (Fig. 1, Supplemental Table 4 (11)) and between testosterone, free testosterone, and testosterone/LH and sperm morphology (Fig. 1, Supplemental Table 5 (11)). These tendencies were more pronounced in the subgroup. Infant serum concentrations of LH and SHBG were not associated with semen quality.

**Table 2.** Total sperm count by reproductive outcome tertiles in infancy. Analyses are presented for the total study population and a subgroup (full-term, not SGA or cryptorchid, and a minipubertal examination at 2.5–3.5 months)

		Total sperm count (million spermatozoa), square-root–transformed							
		Total study population				Subgroup			
	Tertile range	n	Median (IQR)	β (95% CI)	β (95% CI)^a^	n	Median (IQR)	β (95% CI)	β (95% CI)^a^
FSH (U/L)									
T1	(0.09, 0.97)	50	123 (81-256)	Reference	Reference	32	123 (66-216)	Reference	Reference
T2	(0.97, 1.44)	47	119 (40-279)	−1.5 (−3.8-0.8)	−1.8 (−4.1-0.5)	32	105 (40-277)	−1.3 (−4.3-1.6)	−1.8 (−4.9-1.3)
T3	(1.44, 3.99)	49	133 (58-200)	−0.7 (−3.0-1.6)	−1.5 (−3.8-0.9)	32	149 (94-235)	0.0 (−3.0-2.9)	−1.0 (−4.2-2.3)
p_trend_				0.54	0.23			0.98	0.55
Inhibin B (pg/mL)									
T1	(175, 331)	48	151 (66-223)	Reference	Reference	32	149 (72-200)	Reference	Reference
T2	(331, 425)	48	109 (55-186)	−0.8 (−3.1-1.5)	−1.0 (−3.3-1.3)	31	101 (55-174)	−1.0 (−3.9-2.0)	−1.1 (−4.1-2.0)
T3	(425, 695)	47	141 (61-267)	0.4 (−1.9-2.8)	0.2 (−2.1-2.4)	31	200 (58-329)	1.8 (−1.2-4.7)	1.9 (−1.1-4.9)
p_trend_				0.72	0.93			0.25	0.26
Inhibin B/FSH									
T1	(64, 248)	48	159 (91-235)	Reference	Reference	32	153 (94-200)	Reference	Reference
T2	(248, 407)	47	83 (39-190)	−2.3 (−4.6-0.1)	−1.7 (−4.0-0.5)	31	89 (36-235)	−1.9 (−4.9-1.0)	−1.4 (−4.4-1.6)
T3	(407, 4920)	48	126 (83-262)	0.0 (−2.3-2.2)	0.5 (−1.8-2.8)	31	127 (84-265)	0.4 (−2.5-3.4)	1.4 (−1.9-4.6)
p_trend_				0.97	0.71			0.79	0.49
Testis volume (mL)									
T1	(0.06, 0.11)	84	117 (61-219)	Reference	Reference	54	109 (55-257)	Reference	Reference
T2	(0.11, 0.14)	84	147 (67-239)	0.7 (−1.6-2.9)	0.5 (−1.9-2.9)	53	131 (60-202)	0.1 (−3.0-3.3)	0.0 (−3.5-3.4)
T3	(0.14, 0.72)	84	159 (59-298)	1.8 (−0.4-4.0)	1.5 (−0.8-3.9)	54	167 (101-309)	2.5 (−0.6-5.6)	2.2 (−1.3-5.7)
p_trend_				0.11	0.20			0.12	0.22
LH (U/L)									
T1	(0.37, 1.33)	49	127 (82-267)	Reference	Reference	32	126 (63-278)	Reference	Reference
T2	(1.33, 2.11]	48	109 (39-206)	−1.7 (−4.1-0.6)	−0.8 (−3.3-1.7)	32	113 (39-192)	−1.7 (−4.7-1.2)	−1.7 (−5.0-1.5)
T3	(2.11, 5.91)	49	148 (69-218)	−0.1 (−2.4-2.3)	0.1 (−2.3-2.5)	32	159 (89-237)	0.7 (−2.2-3.7)	0.2 (−3.0-3.3)
p_trend_				0.96	0.83			0.63	0.85
Testosterone (nmol/L)									
T1	[0.09, 2.85)	49	84 (54-138)	Reference	Reference	32	84 (40-126)	Reference	Reference
T2	(2.85, 4.11)	49	141 (81-286)	2.3 (0.1-4.6)	2.2 (−0.1-4.5)	32	140 (58-317)	2.9 (0.1-5.8)	3.2 (0.0-6.3)
T3	(4.11, 9.12)	49	193 (56-287)	2.8 (0.5-5.0)	2.3 (0.0-4.7)	32	196 (105-289)	4.1 (1.3-7.0)	3.9 (0.9-7)
p_trend_				0.016	0.052			0.0046	0.012
SHBG (nmol/L)									
T1	(59, 127)	49	109 (55-197)	Reference	Reference	32	95 (47-191)	Reference	Reference
T2	(127, 162)	48	125 (56-262)	0.2 (−2.2-2.5)	−0.5 (−2.8-1.9)	32	125 (41-212)	0.2 (−2.7-3.2)	−0.1 (−3.2-3.0)
T3	(162, 350)	49	168 (58-264)	1.3 (−1.0-3.6)	0.4 (−1.9-2.7)	32	189 (94-288)	2.4 (−0.5-5.4)	1.3 (−1.8-4.3)
p_trend_				0.27	0.72			0.11	0.40
Free testosterone									
T1	(0.0016, 0.0176)	48	118 (61-191)	Reference	Reference	32	120 (53-198)	Reference	Reference
T2	(0.0176, 0.0252)	47	126 (53-294)	0.7 (−1.7-3.0)	0.2 (−2.2-2.6)	31	126 (58-196)	0.1 (−2.9-3.1)	−0.6 (−3.9-2.7)
T3	(0.0252, 0.0521)	48	168 (55-264)	1.9 (−0.5-4.2)	1.9 (−0.4-4.2)	31	190 (47-301)	2.2 (−0.8-5.1)	2.3 (−0.8-5.4)
p_trend_				0.12	0.10			0.15	0.14
Testosterone/LH									
T1	(0.26, 1.56)	48	108 (55-165)	Reference	Reference	32	122 (65-165)	Reference	Reference
T2	(1.56, 2.34)	47	149 (34-289)	1.4 (−0.9-3.8)	1.1 (−1.1-3.4)	31	119 (27-294)	0.8 (−2.1-3.8)	0.5 (−2.7-3.7)
T3	(2.34, 6.90)	48	156 (77-300)	2.3 (0.0-4.7)	1.8 (−0.7-4.2)	31	184 (73-305)	2.4 (−0.6-5.3)	2.2 (−0.9-5.4)
p_trend_				0.049	0.15			0.12	0.16
Free testosterone/LH									
T1	(0.002, 0.009)	48	125 (66-206)	Reference	Reference	32	130 (83-200)	Reference	Reference
T2	(0.009,0.015)	47	89 (34-266)	−0.9 (−3.2-1.5)	−0.2 (−2.4-2.1)	31	125 (27-276)	−0.7 (−3.7-2.3)	−1.1 (−4.3-2.0)
T3	(0.015, 0.045)	48	156 (61-265)	0.9 (−1.4-3.3)	0.5 (−1.8-2.8)	31	127 (47-232)	−0.2 (−3.2-2.8)	0.0 (−3.2-3.2)
p_trend_				0.44	0.69			0.91	0.99
Penile length (cm)									
T1	(2.5, 3.5)	84	168 (74-287)	Reference	Reference	55	152 (75-286)	Reference	Reference
T2	(3.5, 4)	97	109 (53-265)	−1.8 (−3.9-0.4)	−1.1 (−3.3-1.1)	54	107 (45-198)	−2.8 (−5.9-0.4)	−1.8 (−5.2-1.5)
T3	(4.0, 5.0)	70	141 (67-208)	−1.8 (−4.2-0.5)	−1.6 (−4.0-0.9)	51	148 (64-266)	−1.4 (−4.6-1.8)	−0.9 (−4.4-2.5)
p_trend_				0.12	0.20			0.38	0.57

P_trend_: Test for dose-response effect between infancy tertiles and total sperm count. Range per tertile is shown only for the total study population.

Abbreviations: FSH, follicle-stimulating hormone; IQR, interquartile range; LH, luteinizing hormone; SGA, small for gestational age; SHBG, sex hormone binding globulin; T1, lower tertile; T2, middle tertile; T3, upper tertile.

^a^Models were adjusted for maternal smoking [yes, no], maternal diabetes [yes, no], gestational age at birth, weight for gestational age, postnatal age at the infant exam, smoking habits [nonsmoker, occasionally, daily] and total body fat percentage of the young man, and duration of ejaculation abstinence.

Higher FSH in infancy was associated with a lower percentage of progressively motile spermatozoa in adulthood, but this was only statistically significant in the subgroup (Supplemental Table 3 (11)). A similar inverse pattern was observed for inhibin B/FSH. Neither infant FSH nor inhibin B was associated with total sperm count or sperm morphology. Similarly, none of the clinical outcomes in infant boys were significantly associated with semen quality, although there was a nonsignificant tendency toward higher adult total sperm count with higher testis volume in infancy (Table 2). None of the tested associations were significant after multiplicity adjustment (data not shown).

### Associations of Reproductive Hormones and Clinical Outcomes Between Infancy and Adulthood

**Figure 2.**
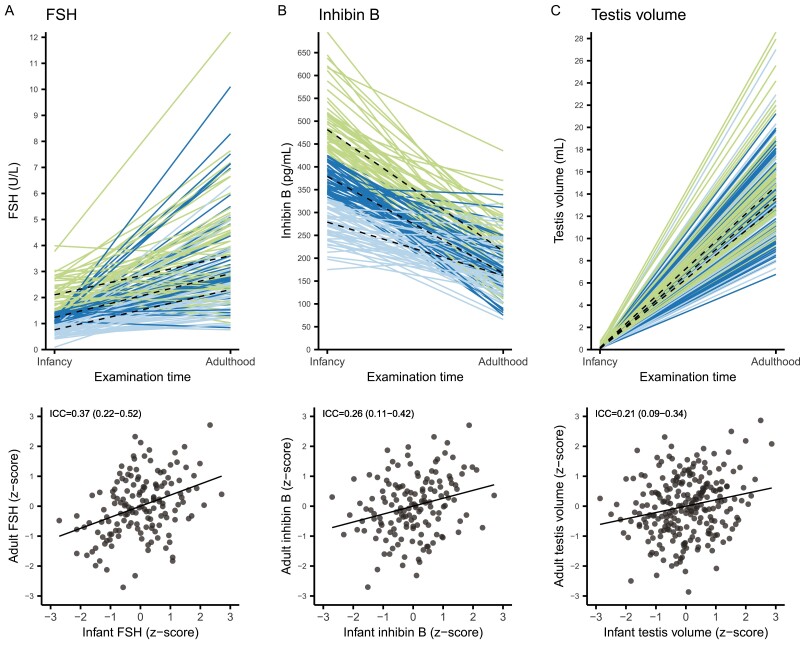
Correlations between reproductive outcomes in infancy and adulthood. Upper panel: Examination time (x-axis) vs measures of reproductive outcomes in infancy and adulthood (y-axis). The dashed lines are the median per tertile in both infancy and adulthood. The colors denote in which tertile each boy is in infancy: green, upper tertile; dark blue, middle tertile; light blue, lower tertile. Lower panel: PP plots of z-scores for reproductive outcomes in infancy (x-axis) vs adulthood (y-axis). The slope of the regression line equals the unadjusted intraclass correlation coefficient, which is denoted in the upper left corner (95% confidence interval). Filled circles are individual observations. Abbreviations: FSH, follicle-stimulating hormone; ICC, intraclass correlation coefficient.

**Figure 3.**
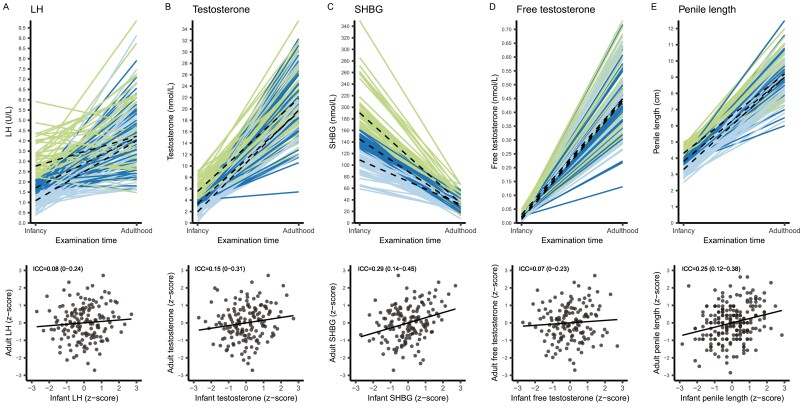
Correlations between reproductive outcomes in infancy and adulthood. Upper panel: Examination times (x-axis) vs measures of reproductive outcomes in infancy and adulthood (y-axis). The dashed lines are the median per tertile in both infancy and adulthood. The colors denote in which tertile each boy is in infancy: green, upper tertile; dark blue, middle tertile; light blue, lower tertile. Lower panel: PP plots of z-scores for reproductive outcomes in infancy (x-axis) vs adulthood (y-axis). The slope of the regression line equals the unadjusted intraclass correlation coefficient, which is denoted in the upper left corner (95% confidence interval). Filled circles are individual observations. Abbreviations: ICC, intraclass correlation coefficient; LH, luteinizing hormone; SHBG, sex hormone binding globulin.

ICCs did not differ substantially between unadjusted and adjusted models (Table 3). ICCs were highest for the FSH/Sertoli cell axis with FSH having the highest ICC of 0.41 (95% CI, 0.26–0.57) in the adjusted model, that is, 41% of the total variation in FSH between infancy and adulthood was explained by the individual’s variation. Thus, a high hormone level at minipuberty was correlated with a high level at adulthood. The ICC for the inhibin B/FSH ratio was comparable to that of FSH (Fig. 2, Supplemental Figure 1 (11)). The ICCs for inhibin B, testis volume, penile length, and SHBG were similar in size, ranging between 0.24 and 0.27 (Table 3, Figs. 2 and 3). ICCs for testosterone, LH, and free testosterone were all ≤ 0.13 and statistically nonsignificant. In the subgroup, ICCs remained generally unchanged, while the ICC for free testosterone/LH almost doubled from 0.19 (95% CI, 0.03–0.36) to 0.41 (95% CI, 0.22–0.59). After adjusting for multiplicity, the ICC for inhibin B in the subgroup was no longer statistically significant, while conclusions on significance levels were the same for all other ICCs (data not shown).

**Table 3.** Intraclass correlation coefficients for reproductive outcomes in infancy and adulthood. Analyses are presented for the total study population and a subgroup (full-term, not SGA or cryptorchid, and a minipubertal examination at 2.5–3.5 months)

	Total study population			Subgroup		
	n	ICC (95% CI)	ICC (95% CI)^a^	n	ICC (95% CI)	ICC (95% CI)^a^
**FSH/Sertoli cell axis**						
FSH	149	0.37 (0.22–0.52)	0.41 (0.26–0.57)	97	0.35 (0.16–0.54)	0.37 (0.17–0.58)
Inhibin B	146	0.26 (0.11–0.42)	0.27 (0.09–0.44)	95	0.22 (0.02–0.42)	0.23 (0.01–0.45)
Testis volume	240	0.21 (0.09–0.34)	0.24 (0.1–0.37)	154	0.24 (0.09–0.4)	0.24 (0.07–0.40)
**LH/Leydig cell axis**						
LH	149	0.08 (0–0.24)	0.07 (0–0.25)	97	0.15 (0–0.35)	0.06 (0–0.28)
Testosterone	150	0.15 (0–0.31)	0.13 (0–0.30)	97	0.20 (0–0.40)	0.16 (0–0.35)
SHBG	149	0.29 (0.14–0.45)	0.24 (0.09–0.39)	97	0.34 (0.15–0.53)	0.27 (0.09–0.46)
Free testosterone	146	0.07 (0–0.23)	0.05 (0–0.23)	95	0.12 (0–0.33)	0.14 (0–0.35)
Penile length	231	0.25 (0.12–0.38)	0.25 (0.12–0.38)	146	0.26 (0.10–0.42)	0.26 (0.08–0.43)
**Ratios**						
Inhibin B/FSH	146	0.39 (0.23–0.54)	0.41 (0.25–0.57)	95	0.39 (0.20–0.58)	0.39 (0.19–0.59)
Testosterone/LH	146	0.25 (0.09–0.41)	0.16 (0–0.32)	95	0.36 (0.16–0.55)	0.29 (0.10–0.47)
Free testosterone/LH	146	0.24 (0.08–0.40)	0.19 (0.03–0.36)	95	0.44 (0.25–0.62)	0.41 (0.22–0.59)

ICCs are interpreted as the fraction of the total variation of each outcome explained by the variation of the individual (e.g., 37% for FSH in the unadjusted model); ICCs where the CI includes zero are considered nonsignificant.

Abbreviations: FSH, follicle-stimulating hormone; ICC, intraclass correlation coefficient; LH, luteinizing hormone; SHBG, sex hormone binding globulin.

^a^Models were adjusted for maternal smoking [yes, no], maternal diabetes [yes, no], gestational age at birth, weight for gestational age, postnatal age at the infant exam, and smoking habits [nonsmoker, occasionally, daily] and total body fat percentage of the young man. In models that included reproductive hormones, time of day for serum sampling was also included.

## Discussion

In this unique prospective birth cohort study spanning roughly 20 years, we investigated associations between pituitary and gonadal hormones in infancy; and markers of testis function in early adulthood in 259 young men from the general population. To our knowledge, this has not been reported before. Interestingly, hormones involving the pituitary-gonadal axis and genital size in 3-month-old boys were associated with semen quality and other markers of testis function in the same individuals at age 19. This suggests a prenatal determination of testis function.

The best infancy predictors of semen quality in the young men were serum testosterone concentrations and the testosterone/LH ratio. This may underline the importance of testosterone for normal development of male reproductive organs early in life (2, 3). Sperm production correlates to the length of the seminiferous tubules. Testosterone may increase tubular length during early infancy by stimulating proliferation of peritubular myoid cells and Sertoli cells, both of which contribute to the tubular lengthening (18). While Sertoli cells do not express androgen receptors at that age, both Leydig cells and myoid cells do (19), and the effects of testosterone might be mediated by other Leydig cell signals and by myoid cell-derived factors (20). Although semen quality is an important factor for a couple’s chances of achieving a pregnancy (21), our study cannot answer the question whether or not reproductive hormone levels in infancy may be markers of future fertility.

While it is well-established that inhibin B and FSH are markers of spermatogenesis in adult men (22), we did not find associations between serum concentrations of inhibin B or FSH in infancy and semen quality in adulthood. However, it is important to remember that the regulation of the feedback mechanisms between the hypothalamus, pituitary, and testis differ between infancy and adulthood (23). We found a nonsignificant tendency toward higher total sperm count in adulthood with higher testis volume in infancy, suggesting that testis volume in infancy may to some degree reflect testicular function similarly to what is known from adult men (24). Sertoli and germ cells proliferate and differentiate during this period (25, 26), but mainly the Sertoli cell population expands. This results in elongation of seminiferous tubules and increased testis volume (9). Therefore, this period shortly after birth is considered an important stage of reproductive development (27).

We found a good correlation between measures of reproductive function in infancy and early adulthood, in particular for the FSH/Sertoli cell axis, indicating that an individual setpoint exists. Notably, although SHBG is generally thought to reflect changes in testosterone to keep a fairly constant serum level of free testosterone, our findings indicate that an individual setpoint also exists for this hormone. Very little is known about SHBG in infancy, although one study found that SHBG levels did not influence serum concentrations of free testosterone in male newborns (28). Oppositely, we found that measurements of testosterone and LH around 3 months of age were not correlated to adult concentrations of these hormones. This may have been due to the fact that we only have one serum sample from each boy in infancy and limited knowledge on onset of and hormone trajectories postnatally, hampering a more precise timing of blood sampling. Studies indicate that both gonadotropins and testosterone in boys increase from around 1 to 2 weeks after birth, peaking at 1 to 3 months (1, 29-31). Around 4 to 6 months postnatally, they decline to prepubertal levels which are often undetectable (32). The range for age at sampling was broad, and we may therefore have sampled the boys in different phases of “minipuberty”, thus underestimating associations. Congruently, correlations became stronger in analyses on the subgroup only, indicating that in the subgroup we may have more precisely sampled at peak time. Conversely, inhibin B increases shortly after birth in infant boys but remains measurable together with FSH throughout childhood (33). Our findings fit well with this as correlations between inhibin B and FSH in infancy and adulthood were generally stronger and did not change in subgroup analyses. Finally, minipubertal timing, intensity, and duration seems to be affected in boys born prematurely (34) and with SGA or cryptorchidism (35) which we also took into consideration in the subgroup analyses by excluding these participants.

A major strength of this study is its longitudinal setup, including a well-characterized population with access to important background information on factors such as maternal lifestyle during pregnancy (5). This is a population-based cohort study with almost 20 years of follow-up time. Participation rate at the adult follow-up was 25%, which is comparable to similar studies. Overall, our population characteristics and reproductive outcomes were comparable to a large contemporary Danish cohort of military conscripts (36). Although ultrasonography and orchidometer assessments of testicular volume correlate well, ultrasound is more accurate than the orchidometer which generally overestimates testicular volume (10). However, our population did not differ from healthy Danish military conscripts on either ultrasound or orchidometer assessed testis volume (36). The young men in our study were generally within the normal weight and body mass index range, and our findings may thus not be applicable to overweight or obese men. The participants in the adult follow-up did not differ significantly from nonparticipants in maternal or fetal parameters. Another strength of the study is the high quality and standardization of outcomes. A minor limitation of the study is that reproductive hormones were measured using immunoassays to enable comparison of previous infancy measurements with adult data, not liquid chromatography–tandem mass spectrometry (LC-MS/MS), which is the gold standard today. This may have added noise to measurements and thus led to an underestimation of associations. However, immunoassay measurements were validated in a subsample of 69 infants from this cohort in whom serum testosterone concentrations were also measured using LC-MS/MS, and the relative difference in measurements between the 2 methods did not change across the measurement range (data not shown).

As this is the first study to explore associations between HPG axis activation in early infancy and adult testicular function, our analyses are to be considered exploratory. We tested many associations with a risk of false positive findings (type I error). While multiplicity adjustment controls the type I error, it inflates the type II error correspondingly. In our study, applying multiplicity adjustment of the *P* values for ICC calculations did not change the conclusions. Models exploring the associations between infant reproductive outcome and adult semen parameters were no longer significant after adjusting for multiplicity.

If essential reproductive programming occurs during fetal life, our results suggest that early infancy may be a “window” to assess prenatal and early life factors that negatively affect male reproduction as such studies otherwise require a very long follow-up period. In conclusion, our present clinical findings support the hypothesis that early postnatal life in boys represents a window to assess future adult reproductive health. Our study is, to the best of our knowledge, the first of its kind and will need corroboration by other long-term cohorts.

## Data Availability

Some or all datasets generated during and/or analyzed during the current study are not publicly available but are available from the corresponding author on reasonable request.

## Abbreviations

DXA: dual-energy x-ray absorptiometry
FSH: follicle-stimulating hormone
GA: gestational age
HPG: hypothalamic-pituitary-gonadal
ICC: intraclass correlation coefficient
IQR: interquartile range
LGA: large for gestational age
LH: luteinizing hormone
LOD: limit of detection
SGA: small for gestational age
SHBG: sex hormone–binding globulin
WGA: weight for gestational age
